# An Economic Evaluation of the TROG 99.03 Trial: Systemic Therapy After Radiotherapy in Early‐Stage Follicular Lymphoma

**DOI:** 10.1002/jha2.70002

**Published:** 2025-02-12

**Authors:** Daniel Erku, Joshua W. D. Tobin, John F. Seymour, Michael MacManus, Paul Scuffham, Greg Hapgood

**Affiliations:** ^1^ Menzies Health Institute Queensland Griffith University Gold Coast Queensland Australia; ^2^ Mater Research Institute University of Queensland Brisbane Queensland Australia; ^3^ Department of Haematology Princess Alexandra Hospital Brisbane Queensland Australia; ^4^ University of Queensland Brisbane Queensland Australia; ^5^ Peter MacCallum Cancer Centre Royal Melbourne Hospital & University of Melbourne Melbourne Victoria Australia

**Keywords:** follicular lymphoma, immunochemotherapy, radiotherapy

## Abstract

**Background:**

The TROG 99.03 trial demonstrated improved progression‐free survival for patients with early‐stage follicular lymphoma (FL) treated with systemic therapy using rituximab‐cyclophosphamide, vincristine, prednisolone (R‐CVP) after involved‐field radiotherapy (RT) versus RT. As systemic therapy was associated with more acute toxicity, the possibility of long‐term toxicity, and no survival benefit yet, the cost‐effectiveness of RT+R‐CVP is important.

**Aim:**

We performed a cost‐effectiveness analysis of RT (reference), RT+CVP, and RT+R‐CVP from the TROG 99.03 trial.

**Methods:**

We constructed a Markov model (15‐year horizon) to compare treatments: RT (reference), RT+CVP and RT+R‐CVP from the 150 patients in the TROG 99.03 trial. Median follow‐up was 11.3 years (range: 4.4–17.8). Lifetime direct health care costs, quality‐adjusted life‐years (QALYs) and incremental cost‐effectiveness ratios (ICERs) were calculated. Australian dollars AUD$50,000 was defined as the proposed willingness‐to‐pay threshold (WTP).

**Results:**

RT+R‐CVP was associated with an improvement of 0.711 QALYs compared to RT, 0.532 QALYs compared to RT+CVP, and was the dominant strategy. The costs of adverse events or retreatment for relapses or transformation had a minimal influence on the ICERs. Sensitivity analyses resulted in ICER values below the WTP with RT+R‐CVP remaining the dominant strategy.

**Conclusion:**

RT+R‐CVP is clearly cost‐effective and was the dominant strategy in early‐stage FL compared to RT or RT+CVP as it delivers superior outcomes at a lower cost from the Australian tax‐payer's perspective.

**Trial Registration:**

The authors have confirmed clinical trial registration is not needed for this submission

## 1 Introduction

Follicular lymphoma (FL) is the most common subtype of indolent non‐Hodgkin lymphoma (NHL). Advanced‐stage FL has a median overall survival (OS) of greater than 15 years but is considered incurable with immunochemotherapy (ICT) [1]. Approximately 20% of patients present with early‐stage FL (Stage I or II) and are treated with curative intent [2]. Radiotherapy (RT) has traditionally been considered the standard of care for early‐stage FL based on studies conducted before the availability of rituximab [3, 4, 5, 6]. As RT is well‐tolerated, provides effective local disease control and may cure a subset of patients [3], it became the standard of care. However, the majority of patients eventually relapse, typically outside the RT field [5, 6].

Registry data suggest the use of front‐line RT alone is declining as treatment shifts to systemic therapy with the availability of rituximab [7, 8, 9]. Rituximab is an anti‐CD20 monoclonal antibody that has contributed to prolonged OS when incorporated into the treatment algorithm in advanced‐stage FL [10]. The use of rituximab‐based ICT in the front‐line setting in early‐stage FL applies the treatment approach commonly used in advanced‐stage FL but long‐term data on its effectiveness is lacking and there is no evidence of the curative potential for such an approach.

The Trans‐Tasman Radiation Oncology Group (TROG) 99.03 trial represents the only modern randomised evidence to guide the treatment of patients with early‐stage FL. It examined the potential utility of systemic therapy to reduce the risk of relapse outside of the RT field. It demonstrated a significantly improved progression‐free survival (PFS) with RT+ICT compared with RT alone [11]. Although this study demonstrates improved PFS, those treated with ICT experienced higher rates of toxicity and with current follow‐up, there is no improvement in OS. Consequently, the use of RT+ICT in front‐line management remains to be universally adopted.

Cost‐effectiveness is an important issue in informed public‐policy decisions [12]. Rituximab has been one of the highest expenditures to the public healthcare system in most developed economies. The Australian Medicare expenditure on biologic medicines in 2015–2016 was approximately Australian dollars (AUD) $2.3 billion dollars, with rituximab being the second highest cost [13]. However, the cost of rituximab in Australia has fallen by approximately 75% since the introduction of biosimilar agents (from $2250 to $562 per dose) [14]. As RT, the standard of care for decades is affordable, the financial burden of the addition of systemic therapy is an important consideration. In the current study, we used the TROG 99.03 trial data to develop a Markov model to assess the cost‐effectiveness of the addition of systemic therapy to RT in early‐stage FL.

## 2 Methods

### 2.1 Patients and Treatment

TROG 99.03 is a randomized, international, multicentre, Phase III trial conducted by the Australasian Leukaemia and Lymphoma Group (ALLG), TROG and Princess Margaret Hospital, Toronto, Canada (ClinicalTrials.gov NCT00115700) [11]. Patients were randomised to either: Arm A, involved field RT alone, or Arm B, identical involved field RT followed by six cycles of systemic therapy. When initially designed, the systemic therapy was the standard regimen of cyclophosphamide, vincristine and prednisolone (CVP). During the course of the trial, practice‐changing RCTs demonstrated improved OS in advanced‐stage FL with the addition of rituximab to chemotherapy [10, 15, 16]. Therefore, systemic therapy was amended to include rituximab in combination with CVP (R‐CVP), in keeping with the evolving standard of care. This amendment also added ^18^F‐labeled fluorodeoxyglucose–positron emission tomography (PET) as it improved staging accuracy in FL and was becoming increasingly available at participating centres [17]. Following therapy, annual CT imaging was mandated for at least 10 years or until disease relapse.

### 2.2 Model Construction

**FIGURE 1:**
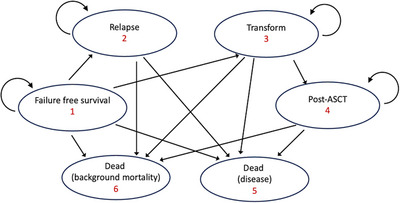
State transition diagram.

A Markov model was developed using TreeAge Pro 2022 software (TreeAge Software, Inc., Williamstown, MA, USA) to analyse the costs and benefits of three front‐line treatment approaches in early‐stage FL: RT alone, RT+CVP, RT+R‐CVP. The Markov model was designed with five distinct health states, each representing a different phase in the progression of the natural history of the disease with treatment (Figure 1). The states were defined as: (1) Failure‐free survival (FFS)—represents patients who remain in ongoing first remission with no requirement for second line treatment; (2) survival with failure—represents patients who developed relapsed or progressive disease and require retreatment; (3) survival with transformation—represents patients who experience clinical suspicion or biopsy‐proven transformation of their FL to aggressive lymphoma; (4) post autologous stem cell transplant (ASCT)—represents patients aged 65 years or younger who experience transformation and undergo an ASCT; (5) death—represented by death from disease or background mortality.

**FIGURE 2:**
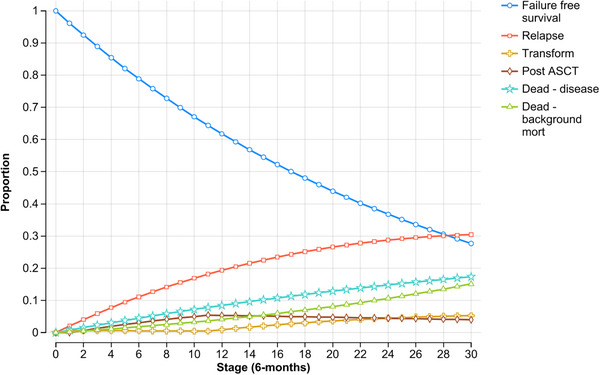
Markov probability analysis for radiotherapy (RT) treatment. The simulation was run for a 15‐year time horizon for each treatment (i.e., 30 cycles). *Stage = cycle length.

**FIGURE 3:**
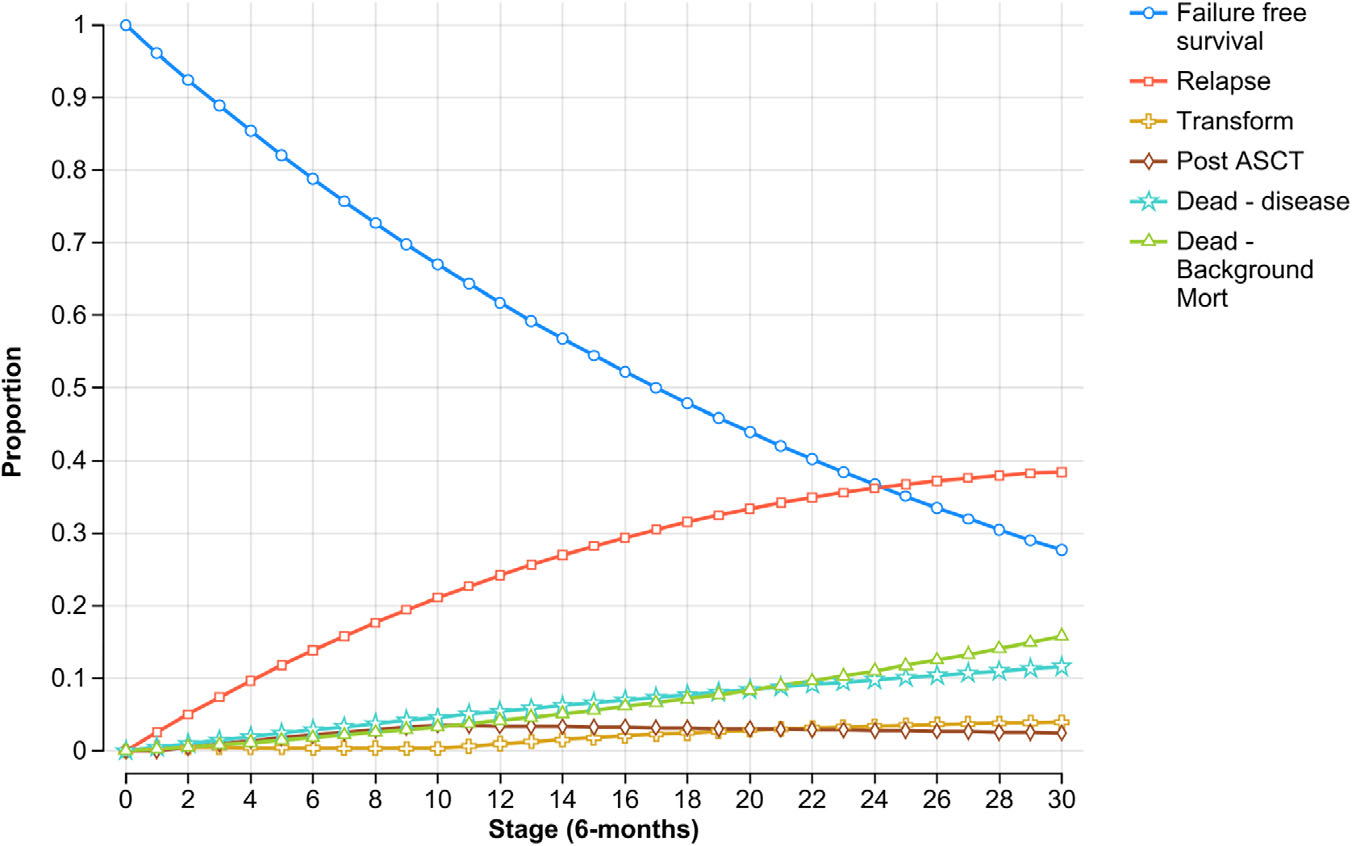
Markov probability analysis for RT+CVP treatment. The simulation was run for a 15‐year time horizon for each treatment (i.e., 30 cycles). *Stage = cycle length.

**FIGURE 4:**
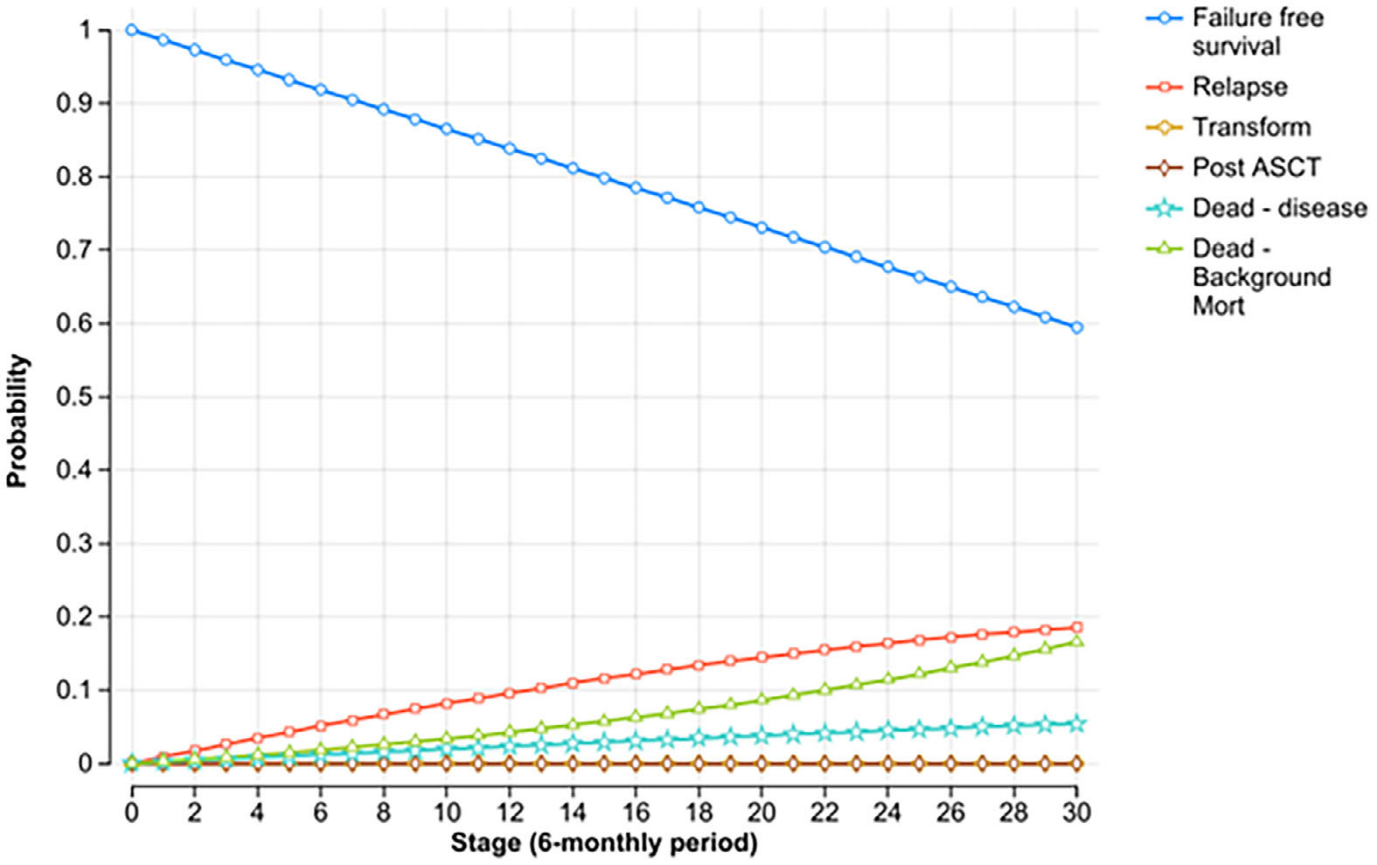
Markov probability analysis for RT+R‐CVP treatment. The simulation was run for a 15‐year time horizon for each treatment (i.e., 30 cycles). *Stage = cycle length.

Once a patient experienced treatment failure (relapse or transformation), within this model they remained in that state for the rest of their life. The probability of transitioning between health states was evaluated on six monthly cycles. Only the first treatment failure was modelled. To ensure uniformity in modelling, each relapse was considered equal to minimise potential variation in second‐line treatment selection and to focus on the primary assessment of FFS among the three treatments. Therefore, all patients at relapse were modelled to receive rituximab, cyclophosphamide, doxorubicin, vincristine, prednisolone (R‐CHOP) treatment. At transformation, all patients received R‐CHOP treatment, and patients under the age of 65 years also received ASCT. The simulation was run for a 15‐year time horizon (i.e., 30 cycles) (Figures 2, 3, 4).

Clinical efficacy and safety/adverse event data were obtained from the TROG 99.03 trial. Transition probabilities, which represent the likelihood of a patient moving from one health state to another over the cycle length, were calculated based on rates (i.e., the number of occurrences of an event for a given number of patients per unit of time). We used a rate‐to‐probability conversion formula to calculate the probability of patients transitioning from one state to another. Background mortality was obtained from the Australian Bureau of Statistics (ABS) life tables, with males aged 50 used as the reference group (Figure S1) [18]. The probability of death from disease and background mortality were modelled separately in two different states to account for the different sources of mortality. It was assumed that adverse drug events were one‐off events during the first cycle of treatment.

Outcomes for the baseline analysis were quality‐adjusted life years (QALYs) and a cost‐utility analysis expressed as an incremental cost‐effectiveness ratio (ICER). Cost‐effectiveness was assessed using the long‐standing willingness‐to‐pay threshold of AUD$50,000 per QALY gained.

### 2.3 Costs and Health State Utilities

An Australian tax‐payer's perspective was adopted to estimate costs of therapy. All costs reflect 2022 values and are reported in Australian dollars (AUD) (AUD$1 ≈ USD$0.67 as of December 2024). The costs of initial FL staging, work‐up and subsequent follow‐up were not included as these were likely to be similar among treatment groups. The costs of therapy and associated administration in the outpatient setting were derived from the Australian Pharmaceutical Benefits Scheme (PBS) and Medicare Schedule (Table 1) [19, 20]. Calculations of a chemotherapy dose and subsequent costs, were based on a patient weight of 70 kg with a body surface area of 1.7 m^2^. The cost of ASCT was derived from the Independent Hospital Pricing Authority [21]. Patients received 15 fractions of RT [22]. The frequencies of common adverse events were derived from the trial data. We estimated costs for Grade 3 or 4 toxicities from current Australian hospital funding resources [23]. An average cost for adverse events was estimated from local activity‐based funding for infection and febrile neutropenia (5‐day admission—$5000), with the cost of pegfilgrastim ($142) modelled for Grade 4 neutropenia.

**TABLE 1:** Cost estimates for front‐line therapy from an Australian tax‐payer perspective.

Treatment regimen	Drug cost per treatment cycle	Related MBS items	Day‐care costs per cycle	Number of cycles	Total cost per cycle	Total cost (all cycles)
Radiation						
Simulation for IMRT	—	15,555	$739.35	1	—	$8238.2
IMRT dosimetry plan	—	15,565	$3448.10	1	—	
Treatment	—	15,275	$190.35	15	—	
Treatment verification	—	15,715	$79.70	15	—	
Systemic therapy						
Rituximab	$562.00	13,921	$110.80	1	$672.8	$672.80
R+CVP	$811.18	13,921	$110.80	6	$921.98	$5531.88
CVP	$249.18	13,921	$110.80	6	$359.98	$2159.88
R‐CHOP	$923.28	13,921	$110.80	6	$1034.08	$6204.48
Adverse events^a^					$5000.00	
Other						
ASCT	—	—	—	—	—	$45,638.63

Abbreviations: ADR, adverse drug events; ASCT, autologous stem cell transplant; BR, bendamustine rituximab; MBS, medicare benefits schedule; IMRT, intensity‐modulated radiation therapy; R‐CHOP, rituximab, cyclophosphamide, doxorubicin, vincristine, prednisolone; R+CVP, rituximab, cyclophosphamide, vincristine, prednisolone; R‐Monotherapy, rituximab monotherapy.

^a^ An average cost was estimated from activity‐based funding for a 5‐day admission for infection and febrile neutropenia.

Since no health‐related quality‐of‐life data were collected in the TROG 99.03 trial, we used utility values of the various health states from previously published studies (Table 2) [24, 25, 26]. We adjusted the utility weights for the cycle length (i.e., 0.5 years) and applied disutility during cycles in which patients received treatment. We also assumed a disutility of 0.2 for the one cycle in which ASCT was performed. The disutility for adverse drug events was already considered in the systemic therapy disutility. In addition, Grade 3 toxicity from infection or febrile neutropenia and Grade 4 neutropenia were included in the model based on their reported incidence in the TROG 99.03 trial. To account for the fact that events could occur at any point within a 6‐month cycle, we applied a half‐cycle correction to all outcomes. All costs and health outcomes were discounted at 5% per annum based on the Australian Pharmaceutical Benefits Advisory Committee (PBAC) guidelines [27].

**TABLE 2:** Quality of life adjustments applied in the model.

Variable	Utility weights	References
Failure free survival utility	0.88	Prettyjohns et al. [24]
Progression without death utility	0.74	Prettyjohns et al. [24]
Radiation disutility	−0.05	Authors' assumption
Systemic therapy disutility	−0.15	Papaioannou et al. [25]
Autologous stem cell transplant disutility	−0.20	Hornberger et al. [26]

### 2.4 Sensitivity Analyses

We performed a series of univariate sensitivity analyses to explore the robustness of the results and evaluate the impact of clinically relevant assumptions. The input variables and assumptions used in the model are presented in the Table S1. For one‐way sensitivity analysis, we tested relevant parameters that had a substantial impact on ICER at the upper and lower limits of plausible ranges (±30% of the base‐case value for probabilities and cost, and ±10% for utilities), and the annual discount rate was varied from 3% to 7%. To determine the effects of uncertainty in all model parameters simultaneously, a Monte Carlo probabilistic sensitivity analysis was performed with 1000 iterations using gamma (γ) distributions for cost and beta (β) distributions for utilities and transition probabilities. All data and analyses are reported in line with the Consolidated Health Economic Evaluation Reporting Standards (CHEERS) criteria (Table S2) [28].

## 3 Results

### 3.1 Patient Characteristics and Outcomes

The economic model was derived from a total of 150 patients with early‐stage FL treated with RT alone (*n* = 75), RT+CVP (*n* = 44) or RT+R‐CVP (*n* = 31) in the TROG 99.03 trial [11]. The median follow‐up for living patients was 11.3 years (range: 4.4–17.8). Baseline patient characteristics are shown in Table S3. The median age was 57 years, 48% of patients were PET‐staged and 75% had Stage I disease. Disease progression occurred in 65 patients: *n* = 38 RT alone, *n* = 22 RT+CVP, *n* = 5 RT+R‐CVP. There were 16 transformation events: *n* = 11 RT alone, *n* = 4 RT+CVP, *n* = 1 RT+R‐CVP. There were 19 deaths: *n* = 13 in RT alone, *n* = 5 RT+CVP, *n* = 1 RT+R‐CVP.

### 3.2 Cost‐Effectiveness Analysis

Cost‐effectiveness outcomes as predicted by the model are presented in Table 3. RT was considered the reference. Using a 5% annual discount rate, the total treatment costs across the time modelled were: RT $43,994, RT+CVP $51,996 RT+R‐CVP $32,043. A total of 7.520 QALYs were expected with RT over 15 years, 7.699 QALYs with RT+CVP and 8.231 with QALYs RT+R‐CVP. Compared to RT alone, RT+CVP resulted in higher costs of $8001 and a gain of 0.18 QALYs producing an ICER of $44,543. RT+R‐CVP compared to RT alone resulted in $11,951 lower costs, 0.711 more QALYs and was a dominant strategy. Comparing RT+R‐CVP to RT+CVP was also a dominant strategy with cost‐savings of $19,952 and 0.532 more QALYs.

**TABLE 3:** Comparison of healthcare costs, quality‐adjusted life‐years and cost‐effectiveness between front‐line treatments.

Strategy	Cost	QALYs	ICER
RT	$43,994	7.520	
RT+CVP	$51,996	7.699	
RT+R‐CVP	$32,043	8.231	
Incremental analysis			
RT+CVP vs. RT	$8001	0.180	44,543
RT+R‐CVP vs. RT	$11,951	0.711	Dominant^a^
RT+R‐CVP vs. RT+CVP	$19,952	0.532	Dominant^a^

Abbreviations: ICER, incremental cost‐effectiveness ratio.

^a^ RT+R‐CVP is the dominant strategy (cost saving and increased QALYs) compared to RT alone and to RT+CVP.

### 3.3 Sensitivity Analysis

**FIGURE 5:**
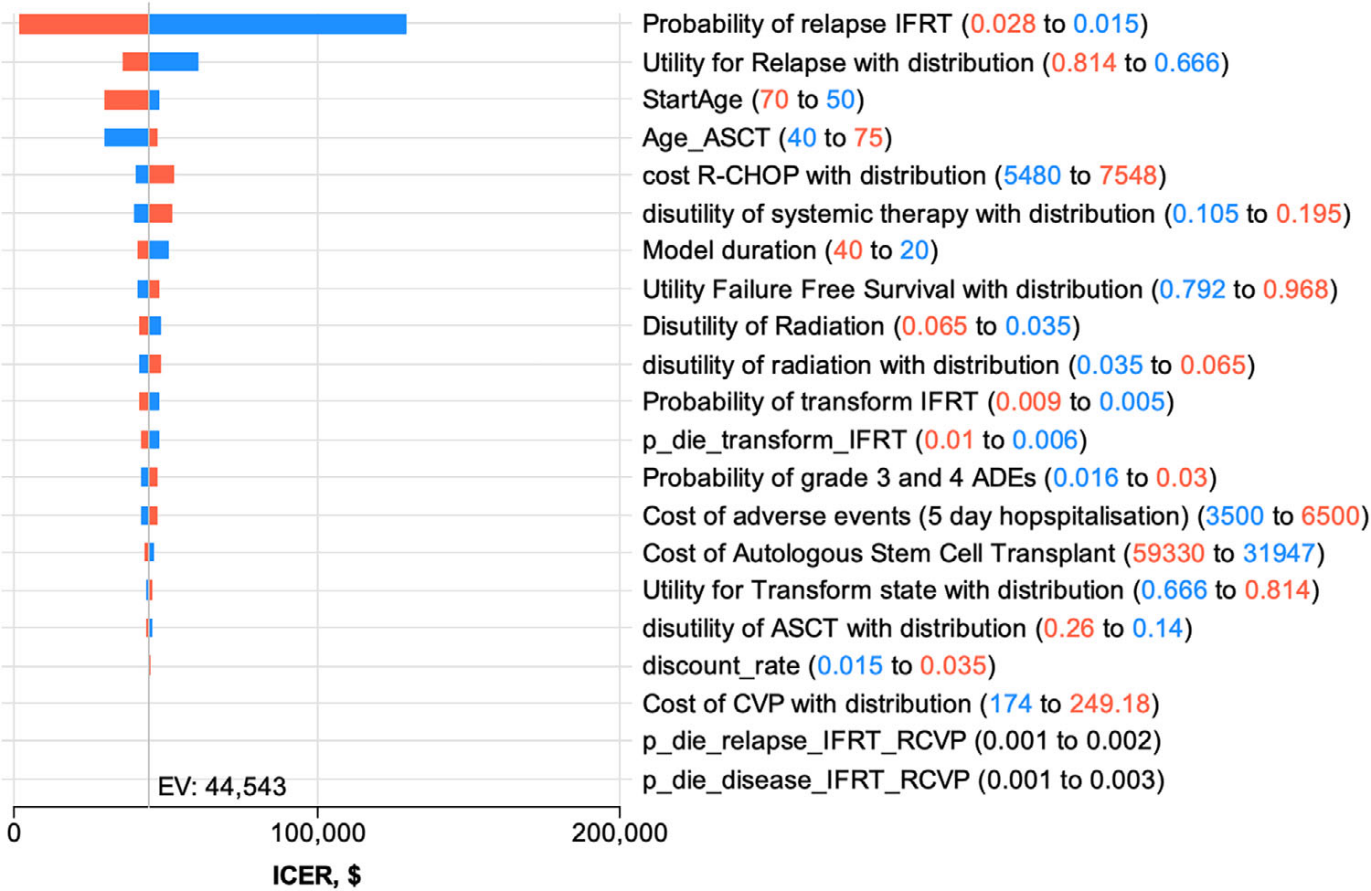
Cost‐effectiveness of RT vs. RT+CVP treatment approach. Tornado diagrams for cost‐effectiveness: RT vs. RT+CVP. Colours are set by parameter range. Red numerals are the upper values and the red bars illustrate the magnitude and direction of the upper value on the ICER. Likewise, blue numerals and blue bars are the lower values (red = high parameter value; blue = low parameter value).

**FIGURE 6:**
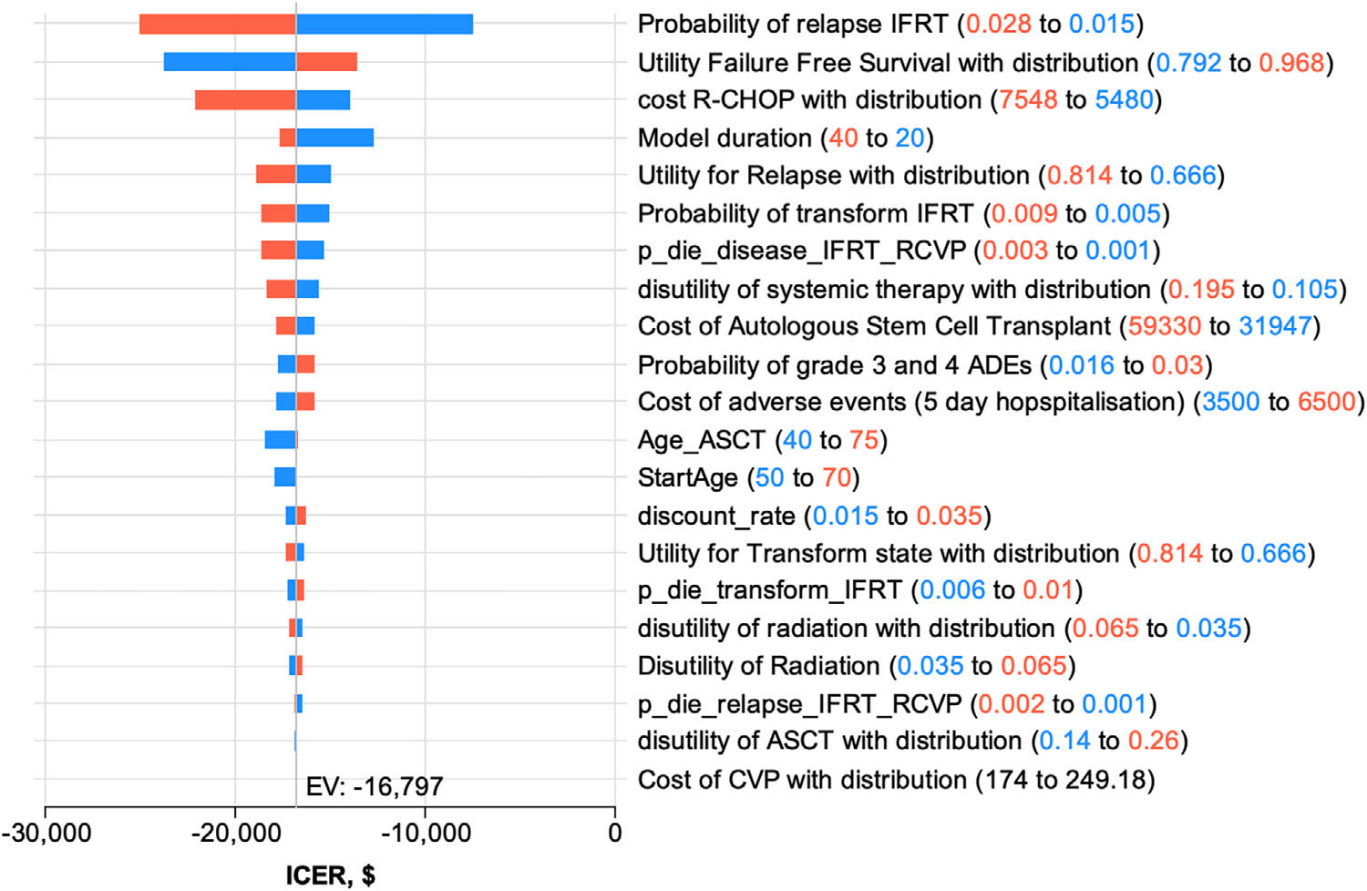
Cost‐effectiveness of RT vs. RT+R‐CVP treatment approach. Tornado diagrams for cost‐effectiveness: RT vs. RT+R‐CVP. Colours are set by parameter range. Red numerals are the upper values and the red bars illustrate the magnitude and direction of the upper value on the ICER. Likewise, blue numerals and blue bars are the lower values (red = high parameter value; blue = low parameter value).

We conducted univariate sensitivity analyses on key variables in the model (Table S4). The tornado diagrams for RT+CVP compared to RT alone (Figure 5) and RT+R‐CVP compared to RT alone (Figure 6) illustrate the sensitivity of the ICERs to changes in those variables. The variables that had the greatest impact on the overall ICERs were the probability of disease relapse in the RT arm, the cost of R‐CHOP and the utility value applied to relapse, and the utility of FFS. We found that all values for these variables tested in the sensitivity analyses resulted in ICER values for RT+R‐CVP below the willingness‐to‐pay threshold of $50,000/QALY gained, with RT+R‐CVP being the dominant strategy (i.e., more effective and less costly than the comparators RT or RT+CVP).

**FIGURE 7:**
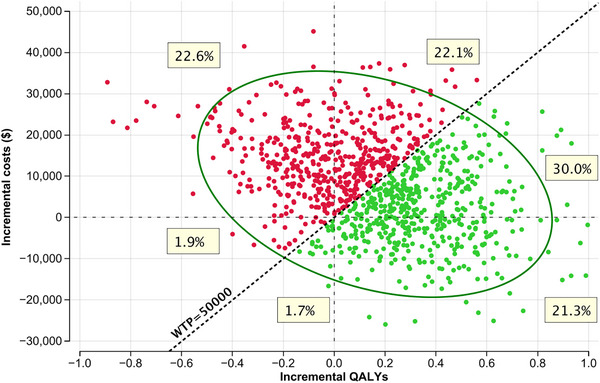
Cost‐effectiveness scatter plot for RT+CVP (comparator) versus RT (baseline). Green dots (RT+CVP) indicate the preferred strategy. 53.7% of simulations are below the $50,000 WTP threshold; 22.6% are better outcomes but at a cost above the $50,000 WTP threshold.

**FIGURE 8:**
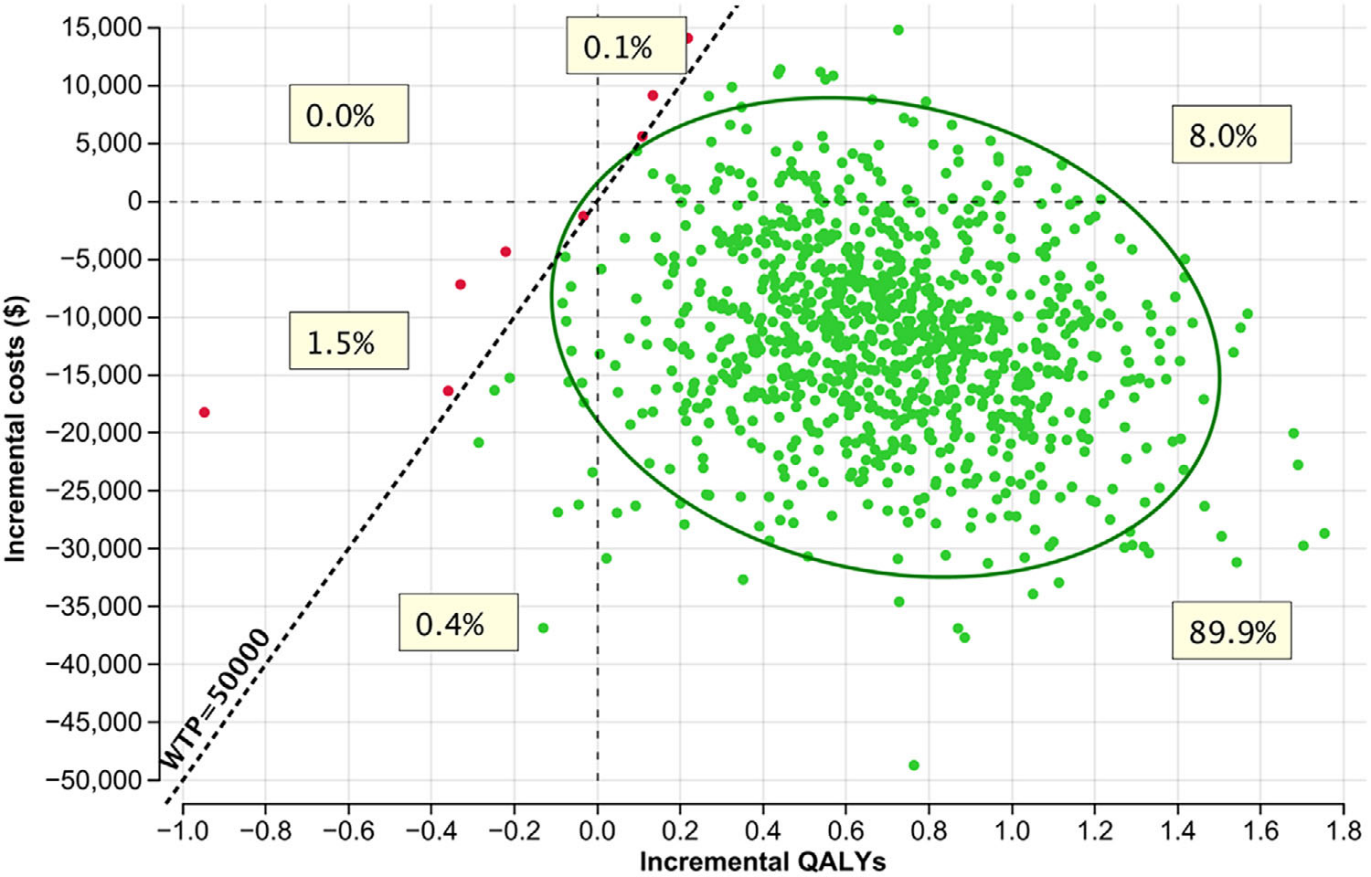
Cost‐effectiveness scatter plot for RT+R‐CVP (comparator) versus RT (baseline). Green dots (RT+R‐CVP) indicate the preferred strategy. 98.3% of simulations are below the $50,000 WTP threshold; 0.1% are better outcomes at a cost above the $50,000 WTP threshold. WTP, willingness‐to‐pay threshold of $50,000.

**FIGURE 9:**
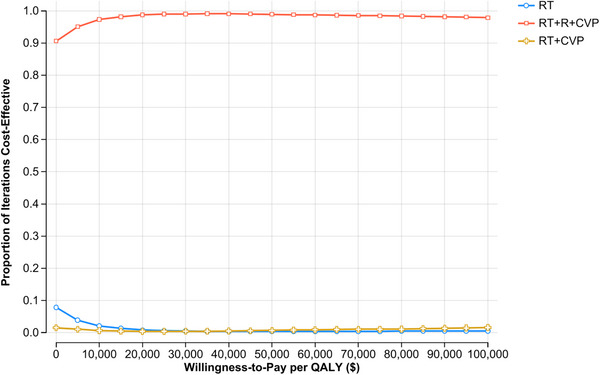
Cost‐effectiveness acceptability curve of the three strategies.

The results of the probabilistic sensitivity analysis are presented in a scatter plots (Figures 7 and 8; Table S5). The scatter plots show the distribution of the incremental costs and QALYs for RT+CVP and RT+R‐CVP compared to RT alone. The majority of the simulated ICER values (> 98% simulations) fell in the lower‐right quadrant of the scatter plot, indicating that RT+R‐CVP is both more effective and less costly than RT alone in most simulations. Cost‐effectiveness acceptability curves were generated for the model (Figure 9).

## 4 Discussion

In this cost‐effectiveness analysis, we analysed if the FFS benefit associated with the addition of systemic R‐CVP therapy to RT alone is cost‐effective in early‐stage FL. Despite the higher upfront direct healthcare costs of RT+R‐CVP, the longer period spent in the FFS state resulted in ICER values below the willingness‐to‐pay threshold due to fewer disease relapses and transformation events leading to lower costs of subsequent therapy. This resulted in substantial savings in the model for RT+R‐CVP in both the base case and sensitivity analyses. The costs of adverse events or retreatment for relapse or transformation (R‐CHOP±ASCT) in these patients had minimal influence in determining the ICER. RT+R‐CVP was the dominant strategy in early‐stage FL compared to RT alone as it delivers superior outcomes at a lower cost from the Australian tax‐payer's perspective.

RT had been the standard of care in early‐stage FL based on retrospective data and a randomised trial conducted prior to the modern era of rituximab and PET‐CT imaging [3, 4, 6, 29, 30]. As most relapses occur outside the RT field [22, 31–34], the concept of adding systemic therapy to RT was explored in the TROG 99.03 trial [11]. The TROG 99.03 trial demonstrated that the addition of systemic therapy to RT improved PFS compared to RT alone but did not show an OS benefit. The patients treated with RT+R‐CVP had a significantly improved PFS compared to patients treated with RT+CVP or RT alone, demonstrating the incremental impact of rituximab. Considering the addition of systemic therapy was associated with more acute toxicity, the possibility of long‐term toxicity and no OS benefit initially shown, assessing the cost‐effectiveness of this strategy is important. Our previous report from the Australasian Lymphoma Alliance of a similar PFS for RT with systemic therapy compared to RT alone differs from the results of the randomised TROG 99.03 trial [35]. This demonstrates the importance of randomised trials to limit selection bias to examine treatment outcomes and the potential for patient characteristics to modify physician treatment selection in retrospective datasets.

Our current modelling demonstrated the longer period spent in the FFS state with RT+R‐CVP resulted in ICER values below the willingness‐to‐pay threshold due to fewer relapses and transformation events. The costs of retreatment for relapse or transformation in these patients were less influential factors in determining the ICER. The base case conclusions of cost‐effectiveness remained unchanged in the sensitivity analyses. RT+R‐CVP was the dominant strategy compared to RT alone and RT+CVP. Interestingly, RT+CVP was cost‐effective (ICER $44,543) compared to RT alone. To our knowledge, there are no utility values specific to early‐stage FL. There were no quality‐of‐life data collected in the TROG 99.03 trial. Therefore, we used utility values from published studies [24, 25, 26], including our prior economic analysis in early‐stage FL [36]. The impact on cost‐effectiveness of both short and long‐term toxicities is often burdened by decreased quality of life, which is reflected in the disutility assigned to both the ‘failure’ health state and the disutility applied in the simulation during treatment periods.

The model was designed to reflect the Australian tax‐payer's perspective in assessing cost‐effectiveness. In Australia, lymphoma treatment is universally available through a public health care system. This enabled the costs of therapy to be obtained from national resources. Although these costs will differ internationally, we believe the results are broadly generalisable due to the time spent in the FFS for patients receiving RT+R‐CVP. The cost of rituximab has fallen significantly since the introduction of biosimilar agents. In Australia, the price has fallen by approximately 75% (from $2250 to $562 per dose) [14]. This price reduction is an important aspect of the cost‐effectiveness of RT+R‐CVP as rituximab is a highly active agent and relatively inexpensive. Although rituximab represents the predominant cost of systemic therapy, varying this through sensitivity analyses did not alter the results.

Our model has several assumptions. Consistent with clinical practice, we assumed that at transformation, patients < 65 years would receive R‐CHOP followed by ASCT, while patients > 65 years would receive R‐CHOP only [37]. We included costs associated with adverse events in two ways. First, disutility for adverse drug events was already considered in the systemic therapy disutility. In addition, we modelled key adverse events (infection, febrile neutropenia, neutropenia) from the TROG 99.03 trial. Only the first treatment failure was modelled and each relapse was considered equal. This was performed to ensure uniformity in modelling to minimise potential variation in second‐line treatment selection and to focus on the primary assessment of FFS among the three treatments. We acknowledge the nature and treatment strategies regarding relapses are variable and that this assumption is a limitation of the study. Conversely, the strengths of this study are the use of randomised clinical trial data that minimizes selection bias compared to retrospective studies, long follow‐up (median: 11.3 years) and the use of annual CT imaging to detect asymptomatic relapse. The protocol amendment for the introduction of rituximab enabled its impact on the disease to be studied.

In conclusion, our data demonstrate RT+R‐CVP was the dominant strategy in early‐stage FL when compared to RT alone or RT‐CVP as it delivers superior outcomes at a lower cost from the Australian tax‐payer's perspective. Consideration of the costs of adverse events, retreatment for transformation or relapse were not influential factors in determining the ICER. The TROG 99.03 trial demonstrates how the use of rituximab with RT has improved the natural history of early‐stage FL. This modelling demonstrates the remarkable economic impact of rituximab that resulted in a dominant strategy, which refers to a cost saving associated with its use. This data will inform future policy and reimbursement decisions.

## Author Contributions

Project design: Daniel Erku, Joshua W. D. Tobin, Paul Scuffham and Greg Hapgood. Contributed data: John F. Seymour, Michael MacManus. Analysed data: Daniel Erku, Joshua W. D. Tobin, Paul Scuffham and Greg Hapgood. Manuscript writing: all authors. All authors approved the final manuscript.

## Conflicts of Interest

John F. Seymour: AbbVie: Advisory board, speakers' bureau, research funding. Astra Zeneca: Advisory board. Beigene: Advisory Board. BMS: Advisory board, speakers' bureau, research funding, expert testimony. Genor Bio: Scientific Advisory Board. Gilead: Advisory Board. Janssen: Advisory board, research funding. Roche: Advisory board, speakers' bureau, research funding, expert testimony. TG Therapeutics: Consultant, expert testimony. The other authors declare no conflicts of interest.

## Supporting information



Supporting Information

## Data Availability

The data from the TROG 99.03 trial that support the findings of this study are available on request and following approval from the Trans‐Tasman Radiation Oncology Group (TROG). The data are not publicly available due to privacy or ethical restrictions.
