# Supplementary material for: An Economic Evaluation of the TROG 99.03 Trial: Systemic Therapy After Radiotherapy in Early‐Stage Follicular Lymphoma
Source: EJHaem. 2025 Feb 12;6(1):e70002. doi: 10.1002/jha2.70002 (PMC11815322; doi:10.1002/jha2.70002)
Supplement: Supplementary file 1 — Supporting Information [file JHA2-6-e70002-s001.docx]

**Supplementary information**

**Table S1.** Input variables for the sensitivity analysis.

| **Variable / Description** | **Mean** | **Low** | **High** | **Distribution** | **SD** | **2.5%** | **97.5%** |
| --- | --- | --- | --- | --- | --- | --- | --- |
| Age for ASCT | 65 | 40 | 75 |  |  |  |  |
| Start age (years) | 60 | 50 | 70 |  |  |  |  |
| Discount rate | 0.05 | 0.03 | 0.07 |  |  |  |  |
| Time horizon (years) | 15 | 10 | 40 |  |  |  |  |
|  |  |  |  |  |  |  |  |
| **Costs** |  |  |  |  |  |  |  |
| Adverse events (5 day hospitalisation) | $5000 | $3500 | $6500 | Gamma | 1448 | 2480 | 8203 |
| CVP | $249 | $174 | $324 | Gamma | 76.7 | 120 | 418 |
| Radiation | $8238 | $5767 | $10710 | Gamma | 2473 | 4134 | 13453 |
| R-CHOP | $6204 | $5480 | $7548 | Gamma | 310 | 5597 | 6833 |
| R-CVP | $5532 | $4487 | $6730 | Gamma | 272 | 4980 | 6051 |
| Autologous Stem Cell Transplant | $45639 | $31947 | $59330 | Gamma | 13835 | 22764 | 78532 |
| Bendamustine + rituximab | $3419 | $2393 | $4445 | Gamma | 1027 | 1664 | 5659 |
| Day-care for systemic therapy | $111 | $78 | $144 | Gamma | 33 | 53 | 184 |
| Rituximab | $673 | $471 | $875 | Gamma | 209 | 342 | 1134 |
|  |  |  |  |  |  |  |  |
| **Transition probabilities** |  |  |  |  |  |  |  |
| Adverse events (grade 3/4) | 0.023 | 0.016 | 0.030 | Beta | 0.0070 | 0.0122 | 0.0400 |
| Neutropenia from chemotherapy | 0.008 | 0.000 | 0.008 | Beta | 0.0025 | 0.0042 | 0.0142 |
| Febrile neutropenia from chemotherapy | 0.002 | 0.000 | 0.002 | Beta | 0.0007 | 0.0009 | 0.0037 |
| Infection from chemotherapy | 0.007 | 0.000 | 0.007 | Beta | 0.0020 | 0.0039 | 0.0118 |
| Death - RT | 0.008 | 0.006 | 0.010 | Beta | 0.0024 | 0.0043 | 0.0138 |
| Death – RT+CVP | 0.005 | 0.003 | 0.006 | Beta | 0.0015 | 0.0027 | 0.0086 |
| Death – RT+R-CVP | 0.002 | 0.001 | 0.003 | Beta | 0.0006 | 0.0011 | 0.0035 |
| Death relapse RT | 0.003 | 0.002 | 0.004 | Beta | 0.0009 | 0.0016 | 0.0052 |
| Death relapse RT+CVP | 0.002 | 0.002 | 0.003 | Beta | 0.0007 | 0.0009 | 0.0037 |
| Death relapse RT+R-CVP | 0.002 | 0.001 | 0.002 | Beta | 0.0005 | 0.0012 | 0.0032 |
| Death transform RT | 0.008 | 0.006 | 0.010 | Beta | 0.0024 | 0.0043 | 0.0138 |
| Death transform RT+CVP | 0.010 | 0.007 | 0.013 | Beta | 0.0030 | 0.0054 | 0.0173 |
| Death transform RT+R-CVP | 0.000 | 0.000 | 0.000 | Beta | 0.0000 | 0.0000 | 0.0000 |
| Relapse RT | 0.021 | 0.015 | 0.028 | Beta | 0.0064 | 0.0112 | 0.0365 |
| Relapse RT+CVP | 0.026 | 0.018 | 0.033 | Beta | 0.0077 | 0.0141 | 0.0445 |
| Relapse RT+R-CVP | 0.009 | 0.006 | 0.012 | Beta | 0.0027 | 0.0048 | 0.0155 |
| Transform RT | 0.007 | 0.005 | 0.009 | Beta | 0.0021 | 0.0038 | 0.0121 |
| Transform RT+CVP | 0.005 | 0.004 | 0.007 | Beta | 0.0016 | 0.0026 | 0.0090 |
| Transform RT+R-CVP | 0.000 | 0.000 | 0.000 | Beta | 0.0000 | 0.0000 | 0.0000 |
|  |  |  |  |  |  |  |  |
| **Utility weights** |  |  |  |  |  |  |  |
| Failure Free Survival | 0.888 | 0.792 | 0.968 | Beta | 0.100 | 0.633 | 0.995 |
| Relapse | 0.745 | 0.666 | 0.814 | Beta | 0.097 | 0.545 | 0.906 |
| Transformation without Death | 0.738 | 0.666 | 0.814 | Beta | 0.101 | 0.525 | 0.901 |
| Disutility Autologous Stem Cell Transplant | 0.201 | 0.140 | 0.260 | Beta | 0.060 | 0.093 | 0.324 |
| Disutility of Radiation | 0.050 | 0.035 | 0.065 | Beta | 0.014 | 0.026 | 0.081 |
| Disutility of Systemic Therapy | 0.148 | 0.105 | 0.195 | Beta | 0.039 | 0.080 | 0.234 |

**Table S2.** Consolidated Health Economic Evaluation Reporting Standards (CHEERS) 2022 checklist^31^.

| **Topic** | **No.** | **Item** | **Location where item is reported** |
| --- | --- | --- | --- |
| **Title** |  |  |  |
|  | 1 | Identify the study as an economic evaluation and specify the interventions being compared. | Title, Page 1 |
| **Abstract** |  |  |  |
|  | 2 | Provide a structured summary that highlights context, key methods, results, and alternative analyses. | Abstract, Page 2 |
| **Introduction** |  |  |  |
| **Background and objectives** | 3 | Give the context for the study, the study question, and its practical relevance for decision making in policy or practice. | Introduction, second and third paragraph |
| **Methods** |  |  |  |
| **Health economic analysis plan** | 4 | Indicate whether a health economic analysis plan was developed and where available. | Methods, Line 9 |
| **Study population** | 5 | Describe characteristics of the study population (such as age range, demographics, socioeconomic, or clinical characteristics). | Methods, Line 1-6 |
| **Setting and location** | 6 | Provide relevant contextual information that may influence findings. | Methods, first Paragraph |
| **Comparators** | 7 | Describe the interventions or strategies being compared and why chosen. | Methods, Third Paragraph |
| **Perspective** | 8 | State the perspective(s) adopted by the study and why chosen. | Methods, sixth Paragraph |
| **Time horizon** | 9 | State the time horizon for the study and why appropriate. | Methods, sixth Paragraph |
| **Discount rate** | 10 | Report the discount rate(s) and reason chosen. | Methods, ninth Paragraph |
| **Selection of outcomes** | 11 | Describe what outcomes were used as the measure(s) of benefit(s) and harm(s). | Methods, Sixth Paragraph |
| **Measurement of outcomes** | 12 | Describe how outcomes used to capture benefit(s) and harm(s) were measured. | Methods, Sixth Paragraph |
| **Valuation of outcomes** | 13 | Describe the population and methods used to measure and value outcomes. | Methods, Sixth paragraph |
| **Measurement and valuation of resources and costs** | 14 | Describe how costs were valued. | Methods, Sixth paragraph |
| **Currency, price date, and conversion** | 15 | Report the dates of the estimated resource quantities and unit costs, plus the currency and year of conversion. | Methods, Seventh Paragraph |
| **Rationale and description of model** | 16 | If modelling is used, describe in detail and why used. Report if the model is publicly available and where it can be accessed. | Methods, Eighth Paragraph |
| **Analytics and assumptions** | 17 | Describe any methods for analysing or statistically transforming data, any extrapolation methods, and approaches for validating any model used. | Methods , Last paragraph and Appendix |
| **Characterising heterogeneity** | 18 | Describe any methods used for estimating how the results of the study vary for subgroups. | Methods , Last paragraph |
| **Characterising distributional effects** | 19 | Describe how impacts are distributed across different individuals or adjustments made to reflect priority populations. | Not applicable |
| **Characterising uncertainty** | 20 | Describe methods to characterise any sources of uncertainty in the analysis. | Methods , Last two paragraphs |
| **Approach to engagement with patients and others affected by the study** | 21 | Describe any approaches to engage patients or service recipients, the general public, communities, or stakeholders (such as clinicians or payers) in the design of the study. | Not reported |
| **Results** |  |  |  |
| **Study parameters** | 22 | Report all analytic inputs (such as values, ranges, references) including uncertainty or distributional assumptions. | Results, first paragraph and Tables |
| **Summary of main results** | 23 | Report the mean values for the main categories of costs and outcomes of interest and summarise them in the most appropriate overall measure. | Results, second paragraph |
| **Effect of uncertainty** | 24 | Describe how uncertainty about analytic judgments, inputs, or projections affect findings. Report the effect of choice of discount rate and time horizon, if applicable. | Results, third paragraph |
| **Effect of engagement with patients and others affected by the study** | 25 | Report on any difference patient/service recipient, general public, community, or stakeholder involvement made to the approach or findings of the study | Not reported |
| **Discussion** |  |  |  |
| **Study findings, limitations, generalisability, and current knowledge** | 26 | Report key findings, limitations, ethical or equity considerations not captured, and how these could affect patients, policy, or practice. | Discussion |
| **Other relevant information** |  |  |  |
| **Source of funding** | 27 | Describe how the study was funded and any role of the funder in the identification, design, conduct, and reporting of the analysis | End of manuscript |
| **Conflicts of interest** | 28 | Report authors conflicts of interest according to journal or International Committee of Medical Journal Editors requirements. | End of manuscript |

**Table S3.** Baseline patient characteristics

**Arm B Arm A**

| **Characteristic** | **Parameter** | **(R)-CVP + IFRT** | **%** | | | **IFRT only** | **%** | |
| --- | --- | --- | --- | --- | --- | --- | --- | --- |
|  |  |  |  | | |  |  | |
| All patients |  | 75 |  | | | 75 |  | |
| Period | Before Rituximab amendment | 44 | 59% | | | 44 | 59% | |
|  | After Rituximab amendment* | 31 | 41% | | | 31 | 41% | |
| Sex | Male | 40 | 53% | | | 38 | 50% | |
|  | Female | 35 | 47% | | | 37 | 50% | |
| Age | Median | 57 |  | | | 57 |  | |
| Stage | 1 | 56 | 75% | | | 57 | 76% | |
|  | 2 | 19 | 25% | | | 18 | 24% | |
| FDG-PET | No | 40 | 53% | | | 38 | 50% | |
|  | Yes | 35 | 47% | | | 37 | 50% | |
| Involved region | Supra-diaphragmatic | 32 | | 42% | 44 | | | 58% |
|  | Infra-diaphragmatic | 43 | | 59% | 30 | | | 41% |
| Bulky disease (>5cm) | No | 65 | | 87% | 64 | | | 85% |
|  | Yes | 10 | | 13% | 11 | | | 15% |
| Extranodal site | No | 68 | | 91% | 70 | | | 93% |
|  | Yes^.^ | 7 | | 9% | 5 | | | 7% |
| Histologic Grade** | 1 | 48 | | 65% | 29 | | | 40% |
|  | 2-3a | 26 | | 35% | 44 | | | 60% |

*All patients randomized to rituximab received it. ** Not recorded for 3 patients

**Table S4.** Univariate sensitivity analysis of cost-effectiveness model

| **Variables** | **Total cost** | | | **Total QALY** | | |
| --- | --- | --- | --- | --- | --- | --- |
|  | **IFRT** | **IFRT_ R+CVP** | **IFRT_CVP** | **IFRT** | **IFRT_**  **R+CVP** | **IFRT_CVP** |
| **Base case** | $43,994 | $32,043 | $51,996 | 7.520 | 8.231 | 7.699 |
| **Utility of failure free survival** |  |  |  |  |  |  |
| 10% decrease in utility | $43,994 | $32,043 | $51,996 | 6.753 | 7.256 | 6.950 |
| 10% increase in utility | $43,994 | $32,043 | $51,996 | 8.159 | 9.044 | 8.324 |
| **Utility of relapse** |  |  |  |  |  |  |
| 10% decrease in utility | $43,994 | $32,043 | $51,996 | 7.330 | 8.130 | 7.461 |
| 10% increase in utility | $43,994 | $32,043 | $51,996 | 7.686 | 8.320 | 7.908 |
| **Utility of progression without death** |  |  |  |  |  |  |
| 10% decrease in utility | $43,994 | $32,043 | $51,996 | 7.500 | 8.231* | 7.684 |
| 10% increase in utility | $43,994 | $32,043 | $51,996 | 7.541 | 8.231* | 7.716 |
| **Disutility of systemic therapy** |  |  |  |  |  |  |
| 10% decrease in disutility | $43,994 | $32,043 | $51,996 | 7.958 | 8.723 | 8.161 |
| 10% decrease in disutility | $43,994 | $32,043 | $51,996 | 7.041 | 7.694 | 7.195 |
| **Cost of R-CVP** |  |  |  |  |  |  |
| 30% decrease in cost | $43,994 | $30,998 | $51,996 | 7.520 | 8.231 | 7.699 |
| 30% increase in cost | $43,994 | $33,241 | $51,996 | 7.520 | 8.231 | 7.699 |
| **Cost of R-CHOP** |  |  |  |  |  |  |
| 30% decrease in cost | $40,110 | $30,185 | $47,312 | 7.520 | 8.231 | 7.699 |
| 30% increase in cost | $51,206 | $35,493 | $60,689 | 7.520 | 8.231 | 7.699 |
| **Cost of ASCT** |  |  |  |  |  |  |
| 30% decrease in cost | $43,254 | $32,043 | $51,508 | 7.520 | 8.231 | 7.699 |
| 30% increase in cost | $44,735 | $32,043 | $52,484 | 7.520 | 8.231 | 7.699 |
| **Cost of grade 3-4 adverse events** |  |  |  |  |  |  |
| 30% decrease in cost | $43,994 | $31,338 | $51,470 | 7.520 | 8.231 | 7.699 |
| 30% increase in cost | $43,994 | $32,749 | $52,521 | 7.520 | 8.231 | 7.699 |
| **Probability of grade 3-4 adverse events** |  |  |  |  |  |  |
| 30% increased prob. | $43,994 | $32,775 | $52,541 | 7.520 | 8.231 | 7.699 |
| 30% decreased prob. | $43,994 | $31,352 | $51,581 | 7.520 | 8.231 | 7.699 |
| **Discount rate** |  |  |  |  |  |  |
| 3% annual rate | $47,205 | $33,730 | $55,908 | 8.022 | 8.800 | 8.218 |
| 7% annual rate | $41,125 | $30,538 | $48,499 | 7.066 | 7.717 | 7.230 |
| **Time horizon** |  |  |  |  |  |  |
| 10 years | $28,725 | $23,878 | $33,507 | 5.676 | 6.059 | 5.770 |
| 20 years | $58,418 | $40,235 | $69,250 | 8.842 | 9.872 | 9.109 |

* There were no cases of progression with RT+R-CVP

**Table S5.** Results from 10,000 Monte Carlo (2^nd^-order) simulations

| **Variable** | **Costs** | | | **Effectiveness** | | |
| --- | --- | --- | --- | --- | --- | --- |
|  | **RT** | **RT+R-CVP** | **RT+CVP** | **RT** | **RT+R-CVP** | **RT+CVP** |
| Mean | $43,720 | $31,950 | $51,474 | 7.532 | 8.244 | 7.710 |
| Std. Deviation | $7,926 | $5,090 | $8,920 | 0.947 | 1.116 | 0.953 |
| Median | $43,448 | $31,620 | $51,041 | 7.679 | 8.462 | 7.848 |
| 95% CI | $29,404 - $60,287 | $22,822 -  $42,779 | $34,913 - $69,776 | 5.295 – 9.006 | 5.524 – 9.829 | 5.465 – 9.180 |

**Figure S1.** Background mortality probability. Calculated using exponential mortality probability (formula in graph) from Australian Bureau of Statistics (ABS) life tables. Males aged 50 were used as the reference group.^18^
